# Block copolymer-assembled nanopore arrays enable ultrasensitive label-free DNA detection†

**DOI:** 10.1039/d4nh00466c

**Published:** 2025-01-17

**Authors:** Maximiliano Jesus Jara Fornerod, Alberto Alvarez-Fernandez, Máté Füredi, Anandapadmanabhan A Rajendran, Beatriz Prieto-Simón, Nicolas H. Voelcker, Stefan Guldin

**Affiliations:** a Department of Chemical Engineering, University College London Torrington Place London WC1E 7JE UK s.guldin@ucl.ac.uk guldin@tum.de; b Materials Physics Center (MPC) - CSICUPV/EHU, Paseo Manuel de Lardizabal 5, Donostia-San Sebastián (Gipuzkoa), 20018 Spain; c Semilab Co. Ltd., Prielle Kornélia u. 2, Budapest, 1117 Hungary; d Department of Electronic Engineering, Universitat Rovira i Virgili Tarragona 43007 Spain; e Institute of Chemical Research of Catalonia, The Barcelona Institute of Science and Technology Av. Països Catalans, 16 Tarragona 43007 Spain; f ICREA Pg. Lluís Companys 23 Barcelona 08010 Spain; g Monash Institute of Pharmaceutical Sciences, Monash University Parkville Victoria 3052 Australia nicolas.voelcker@monash.edu; h Melbourne Centre for Nanofabrication, Victorian Node of the Australian National Fabrication Facility Clayton Victoria 3168 Australia; i Technical University of Munich, Department of Life Science Engineering Gregor-Mendel-Straße 4 Freising, 85354 Germany; j TUMCREATE, 1 CREATE Way, Singapore 138602 Singapore

## Abstract

DNA detection *via* nanoporous-based electrochemical biosensors is a promising method for rapid pathogen identification and disease diagnosis. These sensors detect electrical current variations caused by DNA hybridization in a nanoporous layer on an electrode. Current fabrication techniques for the typically micrometers-thick nanoporous layer often suffer from insufficient control over nanopore dimensions and involve complex fabrication steps, including handling and stacking of a brittle porous membrane. Here, we introduce a bottom-up fabrication process based on the self-assembly of high molecular weight block copolymers with sol–gel precursors to create an inorganic nanoporous thin film directly on electrode surfaces. This approach eliminates the need for elaborate manipulation of the nanoporous membrane, provides fine control over the structural features, and enables surface modification with DNA capture probes. Using this nanoarchitecture with a thickness of 150 nm, we detected DNA sequences derived from 16S rRNA gene fragments of the *E. coli* genome electrochemically in less than 20 minutes, achieving a limit of detection of 30 femtomolar (fM) and a limit of quantification of 500 fM. This development marks a significant step towards a portable, rapid, and accurate DNA detection system.

## Abstract 2

Block copolymer self-assembly enables precise thin-film materials architectures on electrodes for nanopore blockage-based electrochemical DNA detection. This scalable method achieves 30 fM sensitivity in under 20 minutes, advancing portable and accurate biosensing technologies.

## Graphical abstract

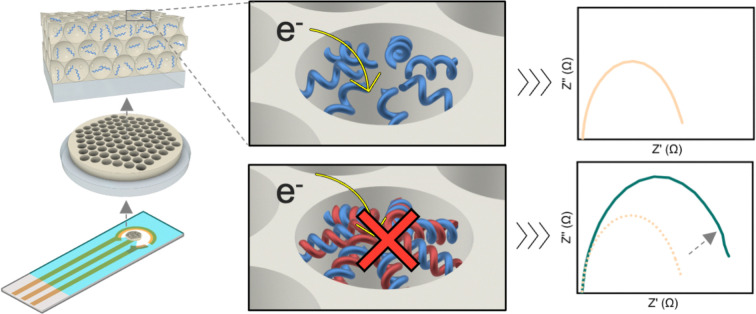

**New concepts**

Despite its critical role in diagnostics, conventional DNA detection methods face limitations in speed, cost, and equipment requirements, hindering their use in point-of-care and resource-limited settings. Electrochemical DNA detection *via* nanopore blockage offers a promising alternative for simple and sensitive biosensing. However, existing material approaches often face challenges such as poor control over the porous nanoarchitecture and cumbersome fabrication methods, which affect reproducibility and scalability. Our work introduces a novel bottom-up fabrication of a nanoporous thin film directly on the working electrode using block copolymer self-assembly. We associate the greatly improved sensor performance to the precise control over nanopore size, uniformity, and arrangement as the structural features are encoded by the design and assembly of the molecular building blocks. The scalability and reproducibility of this solution-based process, combined with its structural tunability for other biomarkers, hold great potential for advancing both fundamental research and practical applications in biosensor development.

## Introduction

Reliable DNA detection is crucial for identifying pathogens, including viruses and bacteria, and in diagnosing a broad spectrum of diseases.^1^ Its importance was highlighted during the COVID-19 pandemic, where it was critical in disease management because it facilitated the identification of infected individuals, thus helping to slow the spread of the disease. DNA detection extends to the diagnosis of non-infectious conditions, such as identifying mutations in circulating cell-free DNA through liquid biopsies or detecting the overexpression of oncogenes in conventional biopsies.^2,3^ Beyond diagnostics, DNA detection finds applications in diverse fields including water treatment evaluation,^4^ environmental and agricultural monitoring,^5^ and biological weapon detection.^6^ From an analytical perspective, polymerase chain reaction (PCR) stands as the gold standard in DNA detection.^7,8^ However, its universal adoption is hindered by time-consuming procedures, the requirement for trained personnel, high materials costs, and the lack of portable equipment.^9–11^

Electrochemical DNA detection offers a compelling alternative to PCR, providing rapid, cost-effective, and labour-free sample preparation with quantitative readouts.^12^ Recent advancements in electrode modification using nanoporous materials have significantly enhanced sensitivity and stability, while reducing the matrix effect in electrochemical (bio)sensors.^13–18^ A major innovation in this field is the use of a nanoporous membrane on top of the electrode, enabling a detection method based on the nanopore blockage (NB) caused upon DNA hybridization.^19–22^ This method relies on variations in the electrochemical signal that occur when target nucleic acids hybridize to their complementary strand inside a nanoscale pore, resulting in pore blockage and a quantitative response that correlates with the concentration of the target analyte.^19,21,23^ Furthermore, tailoring the nanopores size for the target analyte also enhances sensor selectivity by preventing the entry of larger, non-target molecules into the nanopores.^24,25^

Despite notable progress, challenges persist in the production of NB-based DNA sensors. Currently, fabricating these sensors involves mechanically attaching a nanoporous membrane onto a conductive substrate that serves as the electrode. Traditionally, porous silicon (pSi) and nanoporous anodic alumina (NAA) produced *via* electrochemical anodization, have been deployed as the nanoporous layer.^13,26^ However, the inherent fragility of these materials and the complex assembly process of the membrane have limited the application of these DNA sensors beyond research laboratories.^27,28^ Additionally, the production of nanoporous membranes from silicon wafers involves the use of hydrofluoric acid (HF),^14^ a highly toxic substance, while aluminium processing can lead to the formation of potentially explosive by-products.^13^ In light of these challenges, there is an urgent need to develop fabrication methods that enable seamless integration of nanoporous electrode architectures for DNA detection.^16,29^

A promising alternative for producing the nanoporous layer directly on the working electrode involves using block copolymers (BCPs) micelles as sacrificial templates for sol–gel materials.^30^ This bottom-up method enables the creation of inorganic nanopores across the entire mesoporous range (*i.e.* 2 to 50 nm) by using BCPs with varying molecular weights,^31^ and is compatible with a broad spectrum of sol–gel materials.^32,33^ Fine-tuning of nanopore size and porosity is achievable through various processing methods such as solvent vapor annealing,^34^ homopolymer swelling,^35,36^ or chromatographic fractionation of BCPs.^37^ The high level of structural control offered by this fabrication method, along with the ability to directly process this nanomaterial onto the electrode surface without the need for stacking a membrane, holds significant promise for DNA detection. However, the use of sol–gel materials and BCPs in NB-based biosensors remains largely unexplored, mainly due to challenges related to film shrinkage during processing, especially at high block copolymer concentrations, as well as tailored surface functionalization, interfacial reconstruction, and segregation of the sol–gel avoiding continuous pore access towards the bottom electrode.^38,39^

**Fig. 1:**
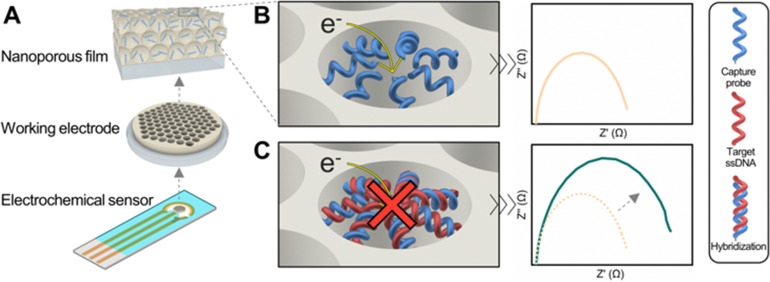
Schematic of the electrochemical DNA detection. (A) The working electrode of a 3-electrode electrochemical biosensor is coated with a block copolymer-derived nanoporous layer and functionalized with ssDNA capture probes designed for selective hybridization of target DNA sequences. (B) Prior to sample exposure, the baseline electrochemical impedance is measured. (C) Hybridization of the DNA sequence in the liquid sample to its matching ssDNA probe on the biosensor results in blocking of the nanopore and an increase in the impedance signal that can be deployed for quantification.

In this work, we report the development of a DNA biosensor based on the NB effect that employs a block copolymer-templated nanoporous thin film as support of the biorecognition layer. We achieved rapid, label-free, and quantitative detection of DNA at femtomolar levels by measuring impedimetric changes resulting from hybridization between single-stranded DNA (ssDNA) capture probes immobilized on the nanopore walls with target ssDNA sequences, corresponding to an *E. coli* genome 16S ribosomal RNA gene fragment (Fig. 1). Characterization of the nanoarchitecture was conducted using various analytical techniques, including spectroscopic ellipsometry, ellipsometric porosimetry, scanning electron microscopy (SEM), focused ion beam (FIB) microscopy, and grazing incidence small-angle X-ray scattering (GISAXS). Additionally, quartz crystal microbalance with dissipation monitoring (QCM-D) enabled to study the efficiency of the functionalization of the nanopores with ssDNA capture probes, and to determine the time required for efficient hybridization.

## Experimental

Reagents: poly(1,4-isoprene)-*block*-poly(ethylene oxide) BCP (polydispersity: 1.01, Mn PI_48_-*b*-PEO_12_ kg mol^−1^) was obtained from polymer source. Toluene (99.9%), toluene (anhydrous, 99.8%), aluminium tri-*sec*-butoxide (97%), 1-butanol (99.4%), (3-glycidyloxypropyl)-trimethoxysilane (GLYMO) (≥98%), (3-aminopropyl)triethoxysilane (APTES) (99%), potassium chloride (KCl) (≥99.9%), glutaraldehyde solution (Grade I, 25% in H_2_O, specially purified for use as an electron microscopy fixative), sodium cyanoborohydride (95%), ethanolamine hydrochloride (≥99.0%), nuclease-free water, and nucleic acids were purchased from Merck. Phosphate buffered saline (PBS) tablets were obtained from OXOID. The electrolytes potassium ferrocyanide K_4_[Fe(CN)_6_] (>98.5%) and potassium ferricyanide K_3_[Fe(CN)_6_] (99+%) were purchased from Honeywell and ACROS Organics, respectively. All chemicals were used as received without further purification. Nucleic acid sequences used for immobilization in the nanoporous structure and sensing are listed in Table 1. Oligos molecular weight (*M*_w_) and melting temperature *T*_m_ were provided by the vendor. NH_2_C6 represents an amine group spaced by six carbons from the DNA bases adenine (A), thymine (T), guanine (G) and cytosine (C). 6FAM and CY5 represent the fluorescent dye modification 6-carboxyfluorescein and cyanine 5, respectively.

**Table 1:** Nucleic acid sequences used for immobilization and sensing

5′ mod	Nucleic acid sequence 5′ to 3′	3′ mod	*M*_w_ (g mol^−1^)	*T*_m_ (°C)
NH_2_C6	GTC CAC GCC GTA AAC GAT GTC GAC TTG G		8769	78.4
NH_2_C6	GTC CAC GCC GTA AAC GAT GTC GAC TTG G	CY5	9302	78.4
NH_2_C6	CAC AAA TTC GGT TCT ACA GGG TA		7227	63.7
	CCA AGT CGA CAT CGT TTA CGG CGT GGA C		8590	78.4
6FAM	CCA AGT CGA CAT CGT TTA CGG CGT GGA C		9127	78.4

Fabrication of nanoporous aluminosilicate thin films directed by block copolymers: two stock solutions with fixed concentrations of aluminosilicate and BCP were prepared. First, the aluminosilicate stock solution was made by mixing a silica precursor (2.8 g of GLYMO), an alumina precursor (0.32 g of aluminium tri-*sec*-butoxide), and 20 mg of KCl in an iced bath. After stirring for 15 min, 135 μl of 10 mM HCl was added dropwise to the blend, followed by another 15 min of stirring at room temperature. Subsequently, 850 μl of 10 mM HCl was added dropwise and stirred for an additional 20 min to complete the hydrolysis. Finally, 2.135 ml of the azeotrope toluene/1-butanol (72.84/27.16 wt%) was added to the solution to get a concentration of 1 g ml^−1^ of aluminosilicate. Next, the solution was filtered with a 0.2 μm polytetrafluoroethylene (PTFE) syringe filter and kept refrigerated at 5 °C for use. Simultaneously, a BCP stock solution was prepared by dissolving 40 mg ml^−1^ of PI-*b*-PEO in the azeotrope toluene/1-butanol.

A so-called hybrid mixture of BCP was created by combining 60 μl of the aluminosilicate stock solution with 750 μl of the BCP stock solution in a glass vial, which was then placed on a shaker for 30 min prior to use.

Thin films were prepared by spin-coating (2,000 rpm, 20 s, Laurell WS 650 MZ) 40 μl of the hybrid solution onto the substrates used for characterization and sensing. These hybrid thin films were subsequently reactive ion etched (2 min, CHF_3_/Ar 15/50, 2 min, 215 W, 40 mbar, PlasmaPro 80 RIE, OXFORD instruments) to remove the upper layer of segregated aluminosilicate aiming to obtain a fully open superficial porous structure, as we have shown in a previous work with enzymes.^39^ Samples were then calcined in argon (450 °C, 30 min, 5 °C min^−1^) in a tubular furnace, left to cool inside the tube and later calcined in air (450 °C, 30 min, 5 °C min^−1^).

The following substrates were used for characterization and sensing. Fluorine-doped tin oxide (FTO)-coated glass (20 × 15 mm^2^, TEC 6, Pilkington) was used as the working electrode for electrochemical DNA sensing. Silica-coated QCM-D sensors (5 MHz 14 mm Cr/Au/SiO2, Quartz PRO) were used for QCM-D measurements. Silicon substrates (10 × 10 mm^2^, 1-side polished, p-type boron, MicroChemicals) were deployed for microscopy, ellipsometric and GISAXS characterization. Gold-coated silicon substrates (10 × 10 mm^2^, Au thickness: 100 nm, coated in an Edwards E306A Bell Jar Thermal Evaporator) were used for FTIR measurements.

Immobilization of ssDNA capture probes on the nanoporous layer: immobilization of the amino-modified capture probes (ssDNA-NH_2_) in the sensors was achieved following a 5-step functionalization procedure. First, nanoporous sensors were plasma-treated in oxygen (15 s, 100 W, 0.33 mbar, Diener Electronic “Pico”) to introduce OH groups on the surface. Second, the sensor surface was aminosilanized by immersing for 20 min in a 5% V/V solution of APTES in anhydrous toluene under an argon atmosphere and room temperature. The functionalized sensors were then sonicated two times for 5 min in toluene and then in ethanol to remove unreacted material from the surface. Third, the sensors were immersed in a solution of 10% V/V of glutaraldehyde in 0.1 M PBS buffer for 30 min at room temperature in air to attach the homobifunctional crosslinker to the amine groups. Nanoporous sensors were then rinsed and sonicated two times for 5 min in PBS to remove unreacted glutaraldehyde molecules. Fourth, modified sensors were incubated overnight at 4 °C in a 1 μM ssDNA-NH_2_ in 0.1 M PBS buffer. The sensor surface was then rinsed three times with a 0.1 M PBS solution. Fifth, the remaining aldehyde groups were blocked with a mixture of 0.1 M ethanolamine and 0.1 M sodium cyanoborohydride in 0.1 M PBS buffer for 30 min. Please note that the PBS buffer used for immobilization and sensing was prepared using nuclease-free water.

### Material characterization

Spectroscopic ellipsometry (SE) and ellipsometric porosimetry (EP): SE and EP measurements were recorded on nanoporous films deposited onto silicon substrates using an ellipsometer with variable angle (incident angle of 73°, spectra: 300 to 989 nm, SE-2000, Semilab). The measured *Ψ* and *Δ* spectra were fitted using the integrated SEA software (Semilab). Refractive index and film thickness were obtained from the experimental data by using a Cauchy dispersion law and Levenberg–Marquardt algorithm (LMA) with a fit quality *R*^2^ > 0.95. Porosity was calculated based on variations in the refractive index resulting from toluene adsorption, utilizing the Lorentz–Lorentz effective medium approximation and a simplex fitting algorithm with a 1e^−6^ error tolerance and up to 1000 iterations.

The distribution of pore sizes was determined using the modified Kelvin equation. It was assumed that the contact angle for toluene on aluminosilicate surfaces is zero, indicating complete wetting.

Grazing-incidence small-angle scattering (GISAXS): GISAXS measurements were completed in a Ganesha 300XL instrument (Xenocs SAXSLAB) on thin films deposited onto silicon substrates. A high-brilliance microfocus Cu-source (*λ* = 1.5418 Å) was used. A PILATUS 300K solid-state photon-counting detector at a sample-to-detector distance of 950 mm and an incidence angle of 0.16° served to collect 2D GISAXS scattering patterns. FitGISAXS^40^ software was used for GISAXS data analysis.

Scanning electron microscopy: SEM images of the aluminosilicate nanoporous films were obtained in an Xbeam 540 FIB/SEM (ZEISS), using short working distance (0.9 to 1 mm) and low acceleration voltage (0.5 to 2 kV). Image analysis was performed with the software WSXM.

Focused ion beam: high magnification image of the nanoporous film surface was captured in a FIB microscope (He, acceleration voltage 25 kV, Orion Nanofab, Carl Zeiss).

Fourier transform infrared spectroscopy: an infrared microscope coupled with an FTIR spectrophotometer (AIM-9000, IRTracer-1000, Shimadzu) was used to record the FTIR spectra in reflection mode on nanoporous thin films fabricated onto gold-coated silicon substrates. The software Lab Solutions IR (Shimadzu) was used to perform atmospheric and baseline corrections.

Quartz crystal microbalance: nucleic acid immobilization and hybridization kinetics were studied with a quartz crystal microbalance (Q-Sense E4 instrument, Biolin Scientific) using flat silica-coated QCM-D sensors (5 MHz 14 mm Cr/Au/SiO_2_, Quartz PRO) and nanoporous-coated QCM-D sensors with an active area of 0.79 cm^2^. A flow rate of 30 μl min^−1^ was set to pump solutions into the QCM-D chamber. Frequency analysis was performed with the software QSense Dfind (Biolin Scientific).

### Electrochemical detection of DNA

Electrochemical measurements: electrochemical measurements were conducted using a three-electrode configuration using a PTFE cell containing the working electrode (exposed area with a diameter of 0.5 cm, FTO coated glass) coated with the nanoporous film functionalized with the capture probes, a silver/silver chloride reference electrode (4 mm diameter, Gamry), and a platinum wire as a counter electrode (0.4 mm diameter, Gamry), as shown in ESI† Fig. S1. Electrochemical impedance spectroscopy (EIS) measurements (100 kHz to 0.1 Hz, amplitude 50 mV, 0 V *vs.* OCP) were carried out using a potentiostat (reference 600 +, Gamry). Electrochemical measurements were analysed and modelled using the Gamry Echem Analyst software.

Electrochemical detection protocol: the working electrodes functionalized with the capture probes (complementary and non-complementary to the target DNA) were mounted in the electrochemical cell. An initial EIS measurement was performed using 500 μl of 2 mM [Fe(CN)_6_]^3/4−^ in PBS buffer, pH 7.4. The sensor was then rinsed with PBS, and a new EIS measurement was performed. This process was repeated until two consecutive EIS measurements were identical to ensure stability in the measurements. Then, 500 μl of the target ssDNA in a concentration from 1 pM to 1 nM prepared in 0.1 M PBS buffer were sequentially incubated on the electrode surface for 20 min. EIS measurements were performed before and after the target ssDNA incubation. EIS measurements with complementary nucleic acid were performed with four sensors. Three sensors were measured with the non-complementary capture probe as a negative control.

## Results and discussion

### Synthesis of inorganic nanoporous films directed by block copolymers

**Fig. 2:**
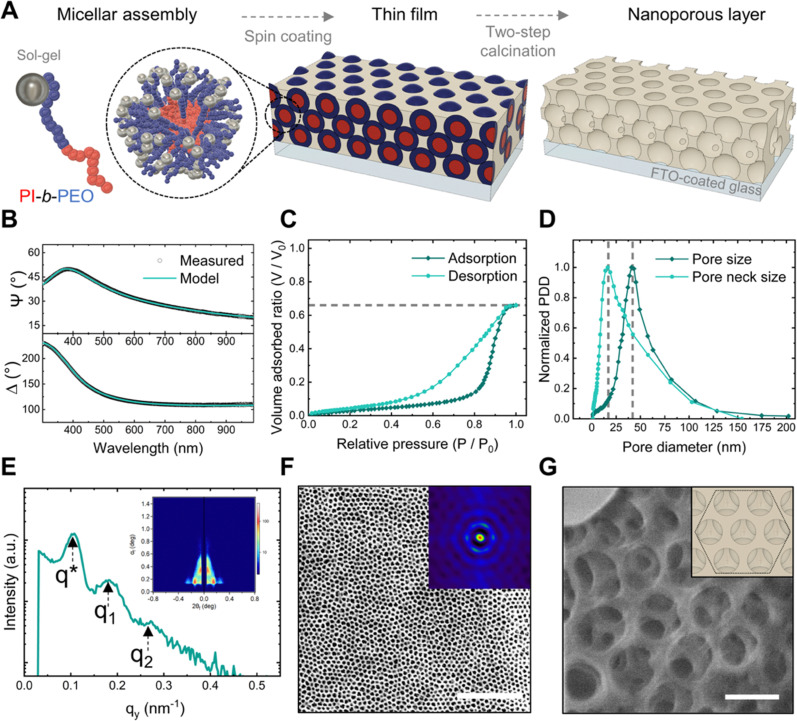
Nanoporous architecture. (A) Schematic of the fabrication process *via* BCP self-assembly. (B) Measured and modelled ellipsometric angles ψ and Δ for film thickness and refractive index determination. (C) Ellipsometric porosimetry isotherms using toluene as adsorptive. (D) Pore size distribution derived from the EP isotherms. (E) 2D GISAXS scattering pattern of the nanoporous film. (F) SEM image of a nanoporous thin film with the 2D spatial distribution function (inset) to evaluate pore ordering. Scale bar: 1 μm. (G) High magnification FIB image showing the nanopores and pore necks alongside a schematic top view of a perfect hexagonal close-packed pore configuration in the inset (FIB image scale bar: 100 nm).

We used the BCP poly(isoprene)-*block*-poly(ethylene oxide) (PI-*b*-PEO) as a structural guide for assembling aluminosilicate sol–gel into a nanoporous architecture, as depicted in Fig. 2A. The initial step involved dissolving PI-*b*-PEO in a selective solvent to induce the self-assembly of the BCP into micelles, and subsequently adding the aluminosilicate sol–gel to co-assemble with the PEO block. This mixture was then spin-coated onto an FTO-coated glass substrate that serves as electrode. The resultant thin film was calcined in two steps: first under argon to condense the sol–gel around the BCP micelles, and then in air to remove the carbonized BCP, revealing the pores.^38^

We used a high BCP concentration in the mixture to generate nanopores with both high porosity and large pore sizes, aiming to facilitate the diffusion of ssDNA within the nanoporous film.^41,42^ The initial calcination step in argon was found in preceding studies to be crucial for mitigating fabrication issues associated with the high BCP content, *i.e.* minimizing uniaxial shrinkage of the sol–gel and preventing collapse of the nanostructure.^38^

We determined the film thickness through spectroscopic ellipsometry (SE) and characterized the accessible porosity, pore dimensions, and surface area *via* ellipsometric porosimetry (EP). A film thickness of approximately 150 nm and a refractive index of 1.12 was derived from fitting the ellipsometric angles ψ and Δ using a Cauchy dispersion law (Fig. 2B). The EP isotherms (dashed line in Fig. 2C) revealed an accessible porosity of 65%. Moreover, the type IV with H2(b) hysteresis loop isotherm suggests the interconnection of the nanopores *via* pore necks.^43^ Applying the modified Kelvin equation on the EP isotherms (Fig. 2D) provided pore size and pore neck size distributions of 44 ± 12 nm and 23 ± 11 nm, respectively.^44^ Additionally, a surface area of 140 m^2^ cm^−3^ was calculated using the Brunauer–Emmett–Teller (BET) method.^45^

To investigate the spatial arrangement of the nanopores, we obtained grazing incidence small-angle X-ray scattering (GISAXS) patterns of the film and high magnification images of the films’ surface. The observation of numerous Bragg peaks in the in-plane line-cuts integration of GISAXS patterns (Fig. 2E) revealed evidence of a long-range porous ordering, with the first Bragg peak (q^*^ = 0.102 nm^−1^) and higher order peaks *q*_1_ and q_2_ consistent with the formation of various symmetric arrangements.^46,47^ The SEM micrograph analysis, by means of a 2D spatial distribution function (inset in Fig. 2F), showed concentric hexagonal rings, indicating a degree of hexagonal close-packed (HCP) order on the nanoporous surface. Additionally, a high magnification focused ion beam (FIB) micrograph (Fig. 2G) confirmed that the surface pores were interconnected with the underlying pores through smaller necks. Alongside, cross-sectional SEM (see micrograph in ESI† Fig. S2) supported the thickness measurements obtained *via* SE.

This nanoarchitecture is a promising candidate for NB-based DNA biosensors due to several reasons. Firstly, its large surface area enables the immobilization of a high density of ssDNA capture probes, thereby increasing the probability and dynamic range for capturing the target DNA molecules. Secondly, the film's thickness is an order of magnitude thinner than that of previously reported NB-sensors.^48^ Prior research indicates that thinner membranes are more effective than thicker ones, as lengthy nanochannels can hinder ion diffusion, leading to increased electrical resistance and reduced sensitivity.^48,49^ Thirdly, the size of the pore necks closely matches that of typical ssDNA capture probes (30 to 40 base pairs in length, equivalent to 10 to 13 nm), potentially enhancing pore-blocking efficiency by aligning the pore diameter with the target molecule size.^49–52^ Finally, we want to highlight that previous NB-based electrochemical biosensors predominantly utilized materials with vertically oriented cylindrical nanopores.^53^

The use of a block copolymer-derived inverse opal-type architecture with highly uniform pore and neck sizes has not been reported yet. We hypothesize that the restrictions imposed by the pore necks could favour pore blocking compared to other configurations. Table 2 summarizes the structural parameters of the nanoporous architecture obtained by SE and EP.

**Table 2:** Structural parameters of the nanostructure

Film thickness [nm]	Porosity [vol%]	Mean pore size *D*_ads_ [nm]	Mean pore neck size *D*_des_ [nm]	Surface area [m^2^ cm^−3^]
150	65	44 ± 12	23 ± 10.5	140

### Surface functionalization with single-stranded DNA capture probes

**Fig. 3:**
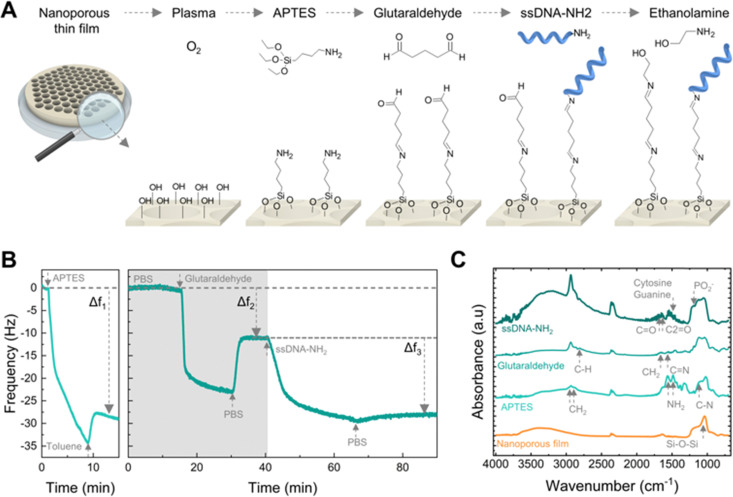
Surface modification with nucleic acid capture probes. (A) Schematic of the surface functionalization with ssDNA. (B) Real-time surface functionalization measurements using QCM-D sensors. Frequency response (5th harmonic) of a sensor coated with a nanoporous film during surface modification in toluene (left) and PBS buffer (right), respectively. (C) FTIR spectra of a nanoporous film during the sequential surface modification with APTES, glutaraldehyde, and amino-modified ssDNA.

We functionalized the nanopore walls with ssDNA capture probes to act as the biorecognition element for targeting specific ssDNA sequences.^54^ The functionalization process involved the sequential use of amino-silane (APTES) and glutaraldehyde (GA) for the immobilization of an amino-modified ssDNA (ssDNA-NH_2_) onto plasma-activated nanopore walls.^55^ Ethanolamine was subsequently applied as a blocking agent of unreacted aldehyde groups. Fig. 3A schematizes the functionalization protocol.

We used a quartz crystal microbalance with dissipation monitoring (QCM-D) to study the surface modification protocol in real-time. Fig. 3B shows the frequency shifts of the fifth harmonic of a QCM-D sensor coated with the nanoporous film during modification with APTES (left) and subsequently with GA and ssDNA-NH_2_ (right). In QCM-D measurements, a decrease in frequency is consistent with mass adsorption at the sensor interface, while frequency increase indicates mass release.^56^ The observed negative frequency shifts upon exposure to APTES, GA, and ssDNA are consistent with the immediate adsorption of these molecules onto the sensor surface. Rinsing with the appropriate solvent aids the removal of non-covalently bound molecules.

Thus, the net negative frequency changes post-rinse (*i.e.*, Δ*f*_1_, Δ*f*_2_, and Δ*f*_3_), observed after each functionalization step, underpin the rapid, stable, and permanent binding of APTES, GA, and ssDNA probes to the surface. We measured the FTIR spectra at each step of the functionalization of the nanoporous surface to confirm the covalent binding (Fig. 3C). The aluminosilicate matrix was identified by the peak at 1037 cm^−1^ corresponding to the asymmetric stretch of Si–O–Si, consistent with the high silica content. Subsequent aminosilanisation led to new peaks at 1550 cm^−1^ and 1485 cm^−1^ attributed to the NH_2_ bending of the amine groups,^57^ as well as the C–N stretching at 1150 cm^−1^ alongside the CH_2_ stretching at 2885 cm^−1^ and 2935 cm^−1^. Next, the crosslinking with glutaraldehyde produced the loss of the NH_2_ bands and the formation of C=N bonds (1652 cm^−1^).^58^ Additionally, the peaks at 1450 cm^−1^ and 2812 cm^−1^ correspond to the CH_2_ deformation and C–H stretching of the aldehyde groups. Attachment of the ssDNA probes produced a peak at 1225 cm^−1^, attributed to the PO_2_^−^ asymmetric stretching of phosphate groups.^59^

Furthermore, DNA base-specific peaks were identified: the peak at 1527 cm^−1^ for the in-plane vibration of cytosine and guanine DNA bases, while the peaks at 1661 cm^−1^ and 1710 cm^−1^ correlated with the C=N stretching in thymine bases and the C=O stretching of guanine groups, respectively.^59^

Additionally, we also verified that ssDNA capture probes were anchored not only on the surface but also within the nanoporous structure by employing ssDNA-NH_2_ modified with the fluorescent molecule cyanine-5 (Cy5) (see ESI,† Fig. S3). The fluorescence intensity of ssDNA functionalized on the nanopore-coated surface was fourteen times greater than that of a flat QCM-D sensor used for reference, confirming that ssDNA was immobilized inside the nanopores and taking advantage of the high surface area available for attachment.

### Electrochemical detection of single-stranded DNA through nanoporous blockage (NB)

**Fig. 4:**
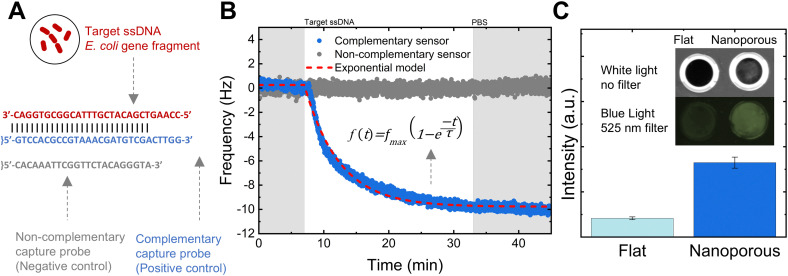
DNA hybridization in the nanoporous layer. (A) DNA sequences of the target and capture probes used for sensing. (B) Frequency changes of nanoporous-coated QCM-D sensors functionalized with complementary and non-complementary capture probes upon exposure to the target ssDNA. (C) Comparative fluorescence intensity between a nanoporous-coated and a flat QCM-D sensors hybridized with a target ssDNA modified with the fluorescent molecule (6FAM) (exposure time: 30 s). The inset shows the QCM-D sensors.

To investigate the use of this material platform for NB-based DNA detection, we employed a 28-base nucleic acid sequence specific to *Escherichia coli* (*E.coli*) and derived from the 16S ribosomal RNA gene as the target ssDNA.^60^ In our sensing approach, we used its complementary ssDNA immobilized within the nanopores as a positive control, alongside a non-complementary ssDNA serving as a negative control, as schematically shown in Fig. 4A. We determined the time required for nucleic acid detection assays by monitoring the frequency changes in nanoporous-coated QCM-D sensors functionalized with the capture probes upon exposure to the target ssDNA (1 nM), as depicted in Fig. 4B.

The frequency variation over time observed in the nanoporous-coated QCM-D sensor functionalized with the complementary capture probe demonstrated the rapid hybridization with the target ssDNA. Conversely, the non-complementary sensor exhibited no lasting frequency changes. We modeled the frequency changes caused by the hybridization between the target ssDNA and its complementary capture probe using an exponential association equation:

1 *f*(*t*) = *f*_max_(1 − exp^−*t*/*τ*^),

where *f*(*t*) represents the frequency change at any given time (*t*), with a time constant *τ* = 5.36 ± 0.63 min (average of three measurements) and *f*_max_ being the maximum frequency change at equilibrium. We established that the optimal time for nucleic acid detection assays is three times the time constant *τ*. This duration represents a balanced compromise between the time necessary for sensing and achieving near equilibrium hybridization (*i.e.* >95%), as we show in the ESI.†

To measure the DNA hybridization efficiency in the nanoporous layer, we used a target ssDNA modified with the fluorescent molecule 6-carboxyfluorescein (6-FAM) and compared the fluorescence intensity between a nanoporous-coated and a flat QCM-D sensor, both functionalized with the complementary ssDNA capture probe (Fig. 4C). The fluorescence intensity of the nanoporous-coated QCM-D sensor was found to be four times greater than that of the flat sensor. This contrasts with the fluorescence intensity previously measured for the capture probes alone, which were more than ten-fold higher on the nanoporous surface compared to the flat sensor. This difference suggests that hybridization predominantly occurs on the surface of the nanoporous layer, effectively blocking access to the underlying nanopores.

We detected the target DNA electrochemically using a three-electrode setup, comprising the FTO-coated glass modified with the nanoporous layer as the working electrode, a platinum wire as the counter electrode, and a silver/silver chloride reference electrode. The redox mediator ferricyanide/ferrocyanide was chosen based on evidence from previous studies that the blocking effect upon DNA hybridization is enhanced with a negatively charged redox probe.^21,22^ See ESI† Fig. S1 for the schematic of the setup.

**Fig. 5:**
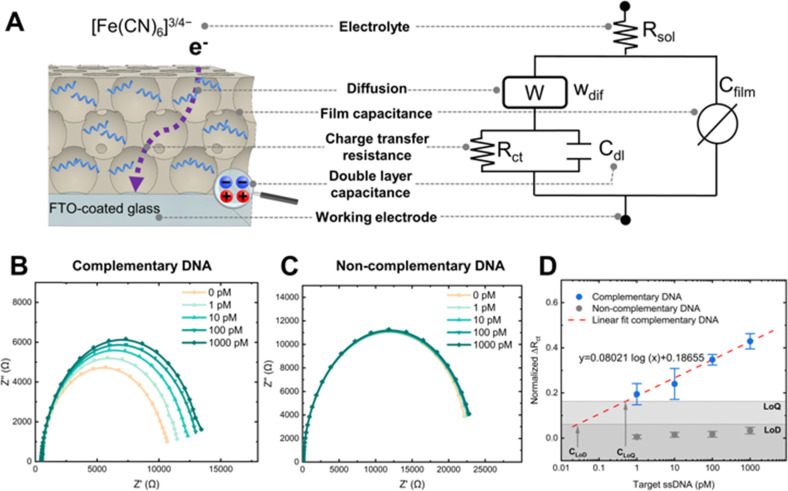
Electrochemical detection of DNA. (A) Schematic and corresponding equivalent circuit of the nanoporous sensor used to interpret impedimetric measurements. (B) Nyquist plots of a complementary sensor (positive control) upon exposure to different concentrations of the target ssDNA. (C) Nyquist plots of a non-complementary sensor (negative control) upon exposure to different concentrations of the target ssDNA. (D) Concentration-response curves of the nanoporous sensors (complementary and non-complementary), error bars correspond to the standard deviation of at least three sensors.

We measured electrochemical impedance spectroscopy (EIS) to monitor changes in the electrical resistance of the system due to nanoporous blockage. An equivalent circuit model of the nanoporous sensor was used to determine changes in electrical resistance from the measured impedance (Fig. 5A).^61^ This model included the charge transfer resistance (*R*_ct_), double-layer capacitance at the electrode interface (*C*_dl_), a Warburg element for diffusion in the film (*W*_dif_), the nanoporous film's capacitance modelled as a constant phase element (*C*_film_), and the solution resistance (*R*_sol_).

Nyquist plots of sensors functionalized with the complementary ssDNA showed distinctive impedance increases upon incubation with target ssDNA, from 1 pM to 1 nM concentrations (Fig. 5B). In contrast, sensors with the non-complementary ssDNA capture probe showed negligible impedance changes (Fig. 5C), proving that the blockage of the nanopores produced the changes in impedance and demonstrating the specificity of the sensor.

We normalized the *R*_ct_ values to compare the response between different biosensors (Fig. 5D). The *R*_ct_ of complementary sensors increased in proportion to the concentration of the target ssDNA, as shown by the linear fit in Fig. 5D. The limit of detection (LoD) is the ability to differentiate a positive result from the noise of a blank measurement.^62^ Thus, a LoD of 30 fM was determined using the mean value of the negative control considering three times its standard deviation, along with the linear fit of the positive control. This LoD represents an improvement of one order of magnitude compared to recent works using carbon-stabilized porous silicon films.^48,63^ A comprehensive comparison of LoD for nanoporous electrochemical systems is presented elsewhere.^28^ Similarly, the limit of quantification (LoQ) is the minimum amount of the target analyte that can be quantified with acceptable precision.^64^ A LoQ of 500 fM was calculated using the average of the negative control increased by ten times its standard deviation,^65^ and the linear fit of the positive control. Please refer to the ESI† for equations used in normalizing the *R*_ct_ and calculating the LoD and LoQ.

The direct fabrication of this nanomaterial onto the working electrode avoids the complex assembly process typical of NB-based biosensors. This advancement, coupled with the successful detection of nucleic acids with an improved LoD compared to previous generations of NB-based biosensors, and the potential for scale-up of the fabrication process, allows envisioning its integration into a test strip for rapid nucleic acid detection similar to those commonly employed for glucose monitoring. Crucially, the capability for quantification extends the relevance of this platform to scenarios requiring the measurement of nucleic acid concentrations. Further improvements in LoDs could be achieved through integration with isothermal nucleic acid amplification techniques, such as loop-mediated isothermal amplification (LAMP) or rolling circle amplification (RCA).^66^ Finally, while the biosensor initially targeted DNA due to its stability and cost-effectiveness compared to RNA, it retains the ability to detect RNA without requiring additional modifications. Direct RNA detection is practical with our current setup since the ssDNA capture probes we used can also hybridize with complementary RNA sequences. However, it's pertinent to mention that the experiments were performed in a simplified buffer system. Despite this, the ultrasensitive fM LoD allows for significant dilution of complex biological samples to levels where the expected DNA and RNA concentrations are usually higher, facilitating detection in clinical and environmental contexts. The coming phase of our research will involve direct comparisons using the equivalent RNA sequences in a complex media to fully validate this capability and expand the scope of our sensor's application.

## Conclusions

In conclusion, this study introduces an electrochemical biosensor fabrication approach based on BCP self-assembly for the rapid, quantitative, selective, ultrasensitive, and label-free DNA detection *via* NB. The deployment of a hexagonal close-packed nanoporous structure, with a thickness of around 150 nm, pores approximately 50 nm in diameter and pore necks around 20 nm, enabled the impedimetric detection of a target ssDNA with a LoD of 30 fM. Moreover, the linear response of the impedimetric measurements, ranging from 1 pM to 1 nM, allowed for quantification with a LoQ of approximately 500 fM. Remarkably, a 20-minute hybridization time is sufficient to achieve near-equilibrium hybridization of complementary DNA strands, demonstrating its potential for rapid sensing.

Nucleic acid detection is fundamental for various applications, including the detection of viruses, bacteria, and disease markers. Nevertheless, the limitations of conventional nucleic acid detection methods prevent their widespread use. The electrochemical biosensor developed in this study presents a substantial advancement towards a portable, user-friendly, rapid, and cost-effective nucleic acid detection platform, providing a viable alternative to current detection technologies.

## Author contributions

M. J. J. F.: conceptualization, methodology, investigation, data analysis, writing – original draft. A. A. F.: methodology, investigation. M. F.: investigation. A. A. R.: methodology, investigation. B. P. S.: conceptualization, supervision. N. H. V.: conceptualization, resources, supervision. S. G.: conceptualization, supervision, resources, project administration, funding acquisition. All authors: writing – review & editing.

## Data availability

Data for this article related to electrochemical measurements, ellipsometry, Fourier Transform Infrared Spectroscopy (FTIR), porosimetry and Quartz Crystal Microbalance with Dissipation Monitoring (QCM-D) are available at ZENODO (DOI: 10.5281/zenodo.13756164).

## Conflicts of interest

M. J. F. and S. G. submitted a patent application (a nanoporous electrode for electrochemical detection of nucleic acids – M. J. Fornerod, S. Guldin, GB2400709.8. Priority date: 18th January 2024) and may be pursuing further commercialisation of the technology.

## Supplementary Material

NH-010-D4NH00466C-s001
