# Supplementary material for: Block copolymer-assembled nanopore arrays enable ultrasensitive label-free DNA detection
Source: Nanoscale Horiz. 2025 Jan 17;10(4):760–9. doi: 10.1039/d4nh00466c (PMC11795167; doi:10.1039/d4nh00466c)
Supplement: NH-010-D4NH00466C-s001 [file NH-010-D4NH00466C-s001.pdf]

Supplementary Information (SI) for Nanoscale Horizons.
This journal is © The Royal Society of Chemistry 2025

-- SUPPORTING INFORMATION --

Block Copolymer-Assembled Nanopore Arrays Enable Ultra-Sensitive Label-Free DNA Detection

Maximiliano Jesus Jara Fornerod[a], *Alberto Alvarez-Fernandez*[a], *Mate Furedi*[a], *Anandapadmanabhan A Rajendran*[b], *Beatriz Prieto-Simon*[c,d], *Nicolas H. Voelcker*[e,f*], *Stefan Guldin*[a,g,*]

[a]Department of Chemical Engineering, University College London, Torrington Place, London, WC1E 7JE, UK.

[b]Department of Electronic Engineering, Universitat Rovira i Virgili, 43007, Tarragona, Spain

[c]Institute of Chemical Research of Catalonia, The Barcelona Institute of Science and Technology, Av. Països Catalans, 16, 43007, Tarragona, Spain

[d]ICREA, Pg. Lluís Companys 23, 08010, Barcelona, Spain

[e]Monash Institute of Pharmaceutical Sciences, Monash University, Parkville, Victoria, 3052, Australia

[f]Melbourne Centre for Nanofabrication, Victorian Node of the Australian National Fabrication Facility, Clayton, Victoria, 3168, Australia

[g]Technical University of Munich, Department of Life Science Engineering, Gregor-Mendel-Straße 4, 85354 Freising, Germany

*corresponding authors. E-mail addresses: nicolas.voelcker@monash.edu, s.guldin@ucl.ac.uk, guldin@tum.de

Keywords:

DNA detection; electrochemical biosensor; nanoporous; block copolymer; sol-gel

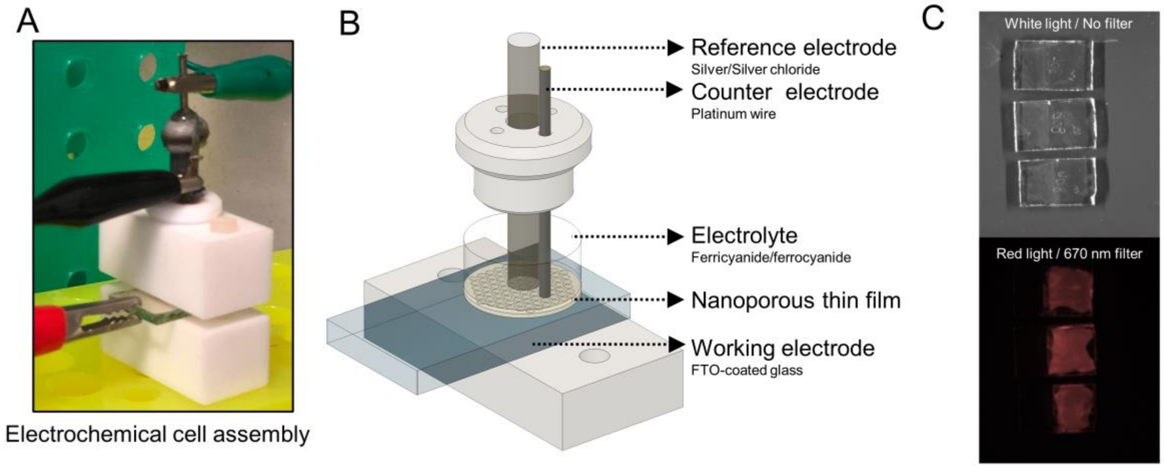

Figure S1: **Electrochemical cell**. A) Photography of the electrochemical cell assembly used for DNA sensing experiments. B) Schematic of the electrochemical cell assembly depicting the internal configuration of the cell. C) Image of nanoporous-coated sensors functionalized with ssDNA-Cy5 under white light (top) and under red light (bottom) (exposure time: 5 s).

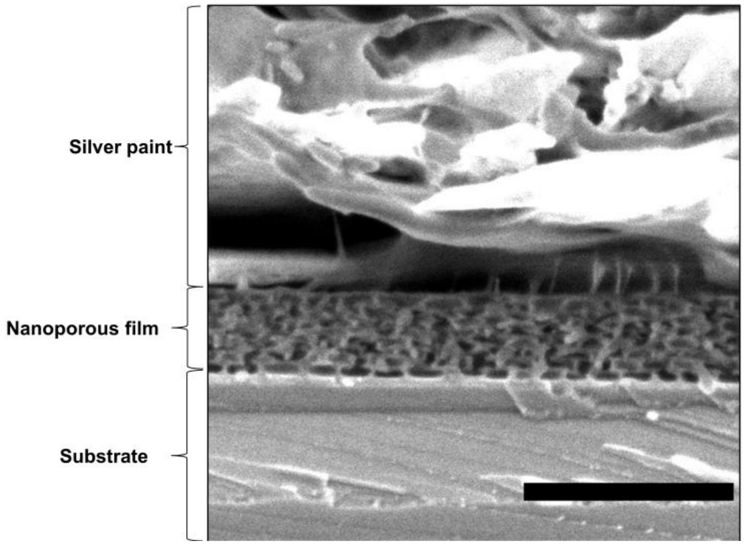

Figure S2: Cross-section of a nanoporous film. SEM image of a sample deposited on silicon substrate (SEM image scale bar: 500 nm).

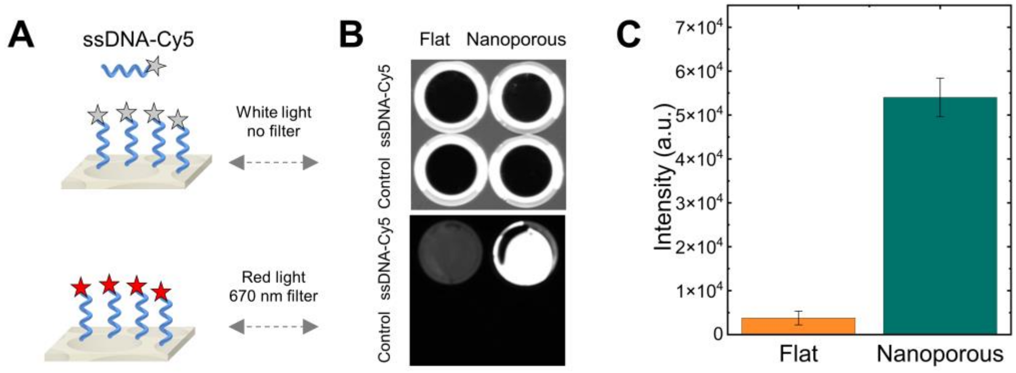

Figure S3: **Surface modification QCM-D sensor using ssDNA-modified with fluorescent tags**. A) Schematic of the nanoporous-coated QCM-D sensors functionalized with ssDNA that was modified with the fluorescent molecule Cyanine5 (Cy5). B) Top view of the modified QCM-D sensors in white light (top) and under red light (bottom) (exposure time: 20s). C) Comparative fluorescence intensity between a flat and a nanoporous QCM-D sensor functionalized with ssDNA-Cy5 (exposure time: 6 s).

1. DNA hybridization time analysis

As shown in the main body of this work the frequency $f(t)$ at a given time (t) can be calculated using the equation (eq. 1):

$$f(t)=f_{max}\left(1-e^{\frac{-t}{\tau}}\right), \quad \text{(eq 1)}$$

where f_{max} denotes the maximum frequency changes at equilibrium for a given concentration of target ssDNA.[1]

Setting the time equal to the time constant, i.e. $t=\tau$, sets equation 1 to:

$$f(t)=f_{max}(1-e^{\frac{-\tau}{\tau}})=f_{max}(1-e^{-1})=f_{max}(0.63),$$

which means that after $t=\tau$ =5.36 min, 63% of the maximum hybridization at equilibrium with the target ssDNA was reached. Please note that this value is independent of f_{max}.

Repeating this analysis for t=2τ, sets equation 1 to:

$$f(t)=f_{max}(1-e^{\frac{-2\tau}{\tau}})=f_{max}(1-e^{-2})=f_{max}\ (0.86),$$

which means after a time $t=2\tau$ =11 min, 86% of the maximum hybridization at equilibrium with the target ssDNA was reached.

Similarly, repeating this analysis and setting hybridization time to t=[3τ; 4τ; 5τ], sets equation 1 to:

$$f(t) = f_{max}\ [0.95; 0.98; 0.99],$$

which means after a time of t=[16; 21; 27] min, 95%, 98%, and 99% of the maximum hybridization at equilibrium with the target ssDNA was reached, respectively.

In summary, the time required to reach the equilibrium f_{max} increases exponentially to infinite. For instance, while it takes 11 min (2τ) to reach 86% of f_{max}, it takes another 10 min to increase this value to 98% (4τ), i.e. in 12 %. Therefore, the hybridization time for nucleic acid detection assays was set to be greater than three times the time constant (i.e. 3τ~16 min), as this represents hybridization near equilibrium (i.e. > 95% f_{max}).

Please note that nucleic acid hybridization is reversible, and f_{max} represents the equilibrium between hybridization and dehybridization reactions.[2] In consequence, f_{max} depends on the target ssDNA concentration. The mathematical analysis suggested that the time (t) required to reach equilibrium (f_{max}) is independent of the value of f_{max}, i.e. it is independent of the target ssDNA concentration. However, hybridization studies have shown that the time constant is inversely proportional to the target DNA concentration.[3] Therefore, the hybridization time determined here provides a timescale for effective sensing with the nanoporous material rather than an exact value for hybridization times due to other variables may also affect this time value, including target concentration, temperature, immobilization on substrates, and formation of secondary structures.[4–6]

2. Charge transfer resistance, limit of detection and limit of quantification

The normalized charge transfer resistance $\Delta R_{ct,\ norm}$ was determined using the charge transfer resistance after incubation with the target ssDNA (R_{ct}^{i}) and the initial charge transfer resistance of the system (R_{ct}^{0}) using the following equation (equation 2):

$$\Delta R_{ct,\ norm}=\frac{R_{ct}^{\ i}-R_{ct}^{\ 0}}{R_{ct}^{\ 0}} \qquad \text{(eq 2)}$$

Equation 3 below shows the linear fit (R^2=0.985) in logarithmic scale that allows quantification of the target ssDNA present in a liquid sample with a slope of the calibration curve representing its sensitivity (0.08 1/pM):

$$\Delta R_{ct,\ norm} = 0.18655 + 0.08021 \log (x), \qquad \text{(eq 3)}$$

where x is the concentration of the target ssDNA in pM.

The LoD was calculated from the values of the negative control by using the following equation (equation 4):

$$LoD = y_{control} + kS_b \qquad \text{(eq 4)}$$

where $y_{control}$ is the average value from 1 pM to 1 nM of the ΔR_{ct} of the negative control, S_b is the standard deviation of the negative control measurements, and k is a numerical factor that is chosen according to the confidence level desired, with a recommended value of 3 for the LoD.[7]

A LoD of 0.0614 ΔR_{ct} was estimated for the DNA sensor, meaning that changes in the ΔR_{ct} smaller than 0.0614 cannot be differentiated from noise with a statistical confidence of 99.7%. The concentration at the LoD (C_{LoD}) was calculated by equalizing the LoD obtained with equation 4 to equation 3, resulting in a C_{LoD} of 30 fM.

LoQ can be calculated similarly to the LoD by using k=10,[8] which resulted in an LoQ of 0.1635 ΔR_{ct}. A corresponding concentration at the LoQ (C_{LoQ}) of 500 fM was calculated by equalizing the LoQ obtained with equation 3.

References

[1] Y. Okahata, M. Kawase, K. Niikura, F. Ohtake, H. Furusawa, Y. Ebara, Anal Chem 70 (1998) 1288–1296.

[2] E. Treasurer, R. Levicky, Journal of Physical Chemistry B 125 (2021) 2976–2986.

[3] S. Xu, J. Zhan, B. Man, S. Jiang, W. Yue, S. Gao, C. Guo, H. Liu, Z. Li, J. Wang, Y. Zhou, Nat Commun 8 (2017) 1–10.

[4] J.X. Zhang, J.Z. Fang, W. Duan, L.R. Wu, A.W. Zhang, N. Dalchau, B. Yordanov, R. Petersen, A. Phillips, D.Y. Zhang, Nat Chem 10 (2018) 91–98.

[5] Y. Gao, L.K. Wolf, R.M. Georgiadis, Nucleic Acids Res 34 (2006) 3370–3377.

[6] T.E. Ouldridge, P. Šulc, F. Romano, J.P.K. Doye, A.A. Louis, Nucleic Acids Res 41 (2013) 8886–8895.

[7] A. Methods Committee, W.H. Evans, D.W. Lord, B.D. RipleyandDr R Wood, with J. J Wilson as Secretary, Analyst 112 (1987) 199–204.

[8] M.E. Zorn, R.D. Gibbons, W.C. Sonzogni, Environ Sci Technol 33 (1999) 2291–2295.
